# Gestational tissue transcriptomics in term and preterm human pregnancies: a systematic review and meta-analysis

**DOI:** 10.1186/s12920-015-0099-8

**Published:** 2015-06-05

**Authors:** Haley R. Eidem, William E. Ackerman, Kriston L. McGary, Patrick Abbot, Antonis Rokas

**Affiliations:** 1 grid.152326.10000000122647217Department of Biological Sciences, Vanderbilt University, VU Station B #35-1634, Nashville, TN 37235 USA; 2 grid.261331.40000000122857943Department of Obstetrics and Gynecology, The Ohio State University, Columbus, OH 43210 USA

**Keywords:** Preterm birth, Gestational tissues, Transcriptomics, Gene expression, microRNA, Methylation, Preeclampsia, Idiopathic preterm birth, Meta-analysis

## Abstract

**Background:**

Preterm birth (PTB), or birth before 37 weeks of gestation, is the leading cause of newborn death worldwide. PTB is a critical area of scientific study not only due to its worldwide toll on human lives and economies, but also due to our limited understanding of its pathogenesis and, therefore, its prevention. This systematic review and meta-analysis synthesizes the landscape of PTB transcriptomics research to further our understanding of the genes and pathways involved in PTB subtypes.

**Methods:**

We evaluated published genome-wide pregnancy studies across gestational tissues and pathologies, including those that focus on PTB, by performing a targeted PubMed MeSH search and systematically reviewing all relevant studies.

**Results:**

Our search yielded 2,361 studies on gestational tissues including placenta, decidua, myometrium, maternal blood, cervix, fetal membranes (chorion and amnion), umbilical cord, fetal blood, and basal plate. Selecting only those original research studies that measured transcription on a genome-wide scale and reported lists of expressed genetic elements identified 93 gene expression, 21 microRNA, and 20 methylation studies. Although 30 % of all PTB cases are due to medical indications, 76 % of the preterm studies focused on them. In contrast, only 18 % of the preterm studies focused on spontaneous onset of labor, which is responsible for 45 % of all PTB cases. Furthermore, only 23 of the 10,993 unique genetic elements reported to be transcriptionally active were recovered 10 or more times in these 134 studies. Meta-analysis of the 93 gene expression studies across 9 distinct gestational tissues and 29 clinical phenotypes showed limited overlap of genes identified as differentially expressed across studies.

**Conclusions:**

Overall, profiles of differentially expressed genes were highly heterogeneous both between as well as within clinical subtypes and tissues as well as between studies of the same clinical subtype *and* tissue. These results suggest that large gaps still exist in the transcriptomic study of specific clinical subtypes as well in the generation of the transcriptional profile of well-studied clinical subtypes; understanding the complex landscape of prematurity will require large-scale, systematic genome-wide analyses of human gestational tissues on both understudied and well-studied subtypes alike.

**Electronic supplementary material:**

The online version of this article (doi:10.1186/s12920-015-0099-8) contains supplementary material, which is available to authorized users.

## Background

In humans, gestation typically lasts 40 weeks; preterm birth (PTB) is defined as birth before 37 completed weeks of gestation and is the leading cause of newborn death worldwide. More than 15 million babies are born too soon every year and rates of PTB had been increasing until 2006 when changes in obstetrical practices regarding early cesarean sections led to a recent decrease in deliveries before term [1]. Nevertheless, 10 % of pregnancies still end before 37 weeks across the world and this high incidence of PTB is problematic because premature babies are at higher risk for lifelong health and developmental problems [2, 3]. For example, almost half of all children born premature suffer from vision or hearing loss and learning disabilities at some point in their life [4, 5]. The combined medical costs stemming from care during the labor and delivery process as well as from care later in life are estimated to be near $26 billion annually [6].

PTB is a complex, multifactorial syndrome comprised of multiple clinical subtypes, which often occur at different gestational ages and can be defined as either ‘spontaneous’ or ‘medically indicated’ [7]. Medically indicated preterm deliveries account for 30 % of PTB cases and are often preceded by complications including preeclampsia (PE), intrauterine growth restriction (IUGR), gestational diabetes mellitus (GDM), and chorioamnionitis [8]. The remaining 70 % of PTB cases are idiopathic; 45 % is due to the spontaneous onset of labor (sPTB) and the remaining 25 % is due to the preterm premature rupture of membranes (PPROM) [9–11]. Regardless of PTB subtype, however, current therapies are not successful in prolonging time to birth once labor has been initiated and the most effective therapy, progesterone supplementation, is only effective in a small number of high-risk cases [12]. It is critical that we gain greater insight into the genes and pathways that regulate birth timing in humans in order to develop effective prevention and treatment strategies, including for cases of sPTB.

A number of environmental risk factors have been associated with sPTB including infection, nutrition, socioeconomic status, and stress but the pathways through which these risk factors act remain unclear [13]. Recent evidence from family, twin, and case–control studies suggests that genetics also plays an important role in birth timing, and the heritability of PTB is estimated to be approximately 30 % [1, 6, 8]. Thus, PTB tends to run in families and women who were born preterm are also more likely to deliver preterm themselves. Interestingly, however, fathers born prematurely do not appear to pass on this risk to offspring [1]. Furthermore, one of the strongest predictors of PTB is previous preterm birth and, in subsequent pregnancies from the same woman, birth timing tends to occur around the same gestational age for each pregnancy [9, 14]. Candidate gene studies have targeted genes with known biological roles potentially related to processes occurring during pregnancy but, in general, teasing apart the complex genetic architecture of pregnancy and PTB has proved challenging.

**Fig. 1:**
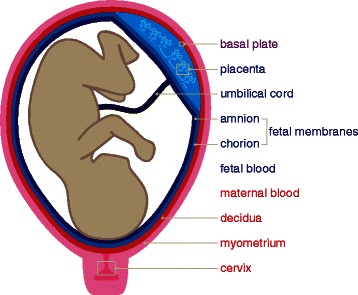
The tissues of pregnancy. Our systematic literature review surveyed a total of 9 distinct gestational tissue types including 4 of maternal origin (cervix, myometrium, decidua, and maternal blood; shown in red), 4 of fetal origin (fetal blood, fetal membranes, umbilical cord, and placenta; shown in blue), and 1 of mixed maternal and fetal origin (basal plate; shown in purple)

Further complicating our understanding of PTB genetic architecture are the numerous maternal and fetal gestational tissues that must interact to facilitate parturition [12, 15]. These tissues include decidua, myometrium, cervix and maternal blood originating from the mother and villous placenta, fetal membranes (chorion and amnion), umbilical cord, and fetal blood originating from the fetus (Fig. 1). Furthermore, the basal plate is a region at the maternofetal interface that is commonly biopsied for the study of PTB and includes cells from both the decidua and villous placenta. The decidua, myometrium, and cervix act to house the fetus as well as expel it during labor and delivery, the chorion and amnion act as membranes separating the fetus from the mother, and the umbilical cord allows for efficient nutrient transfer. Together, these tissues share a general functionality in the efficient maternofetal exchange of nutrients, gas, and waste.

Although little is known about the complex etiology of PTB, many studies have generated pregnancy-related transcriptomes in various tissue types and pathologies. Because of the diversity of tissues and clinical subtypes involved as well as the large number of questions examined, few studies have attempted to synthesize any dimension of the admittedly complex transcriptional landscape of this multifactorial syndrome. To synthesize what is known about PTB transcriptomics, we analyzed all published genome-wide studies of gestational tissues (placenta, decidua, myometrium, maternal blood, cervix, basal plate, fetal membranes, umbilical cord, and fetal blood) in both healthy and diseased human pregnancies to identify all statistically supported candidate genetic elements in PTB subtypes.

Our meta-analysis identified 134 genome-wide studies of pregnancy and PTB. The majority of PTB research focused on PE; very few studies were focused on sPTB (18 %) even though sPTB accounts for 45 % of all PTB cases. Moreover, there was limited overlap in the identity of candidate genes across studies. In placenta (n = 53), for example, 6,444 differentially expressed unique genes were identified but only 2, *LEP* and *FLT1*, were present in more than 10 gene expression studies. Similarly, in PE studies (n = 27), 5,329 differentially expressed unique genes were identified but only 13 were found in 5 or more gene expression studies. The limited overlap of differentially expressed genes across studies of the same tissue or clinical subtype as well as the highly uneven coverage of studies targeting highly prevalent clinical subtypes suggest that larger-scale, systematic studies aimed at understanding the transcriptional profiles of the diverse clinical PTB subtypes and characterizing their disease-relevant transcriptional differences will be necessary to identify genes whose dysregulation contributes to this complex, multifactorial syndrome.

## Results

### A systematic review identified 134 transcriptomic studies on 9 gestational tissues and 29 different phenotypes

Of the 2,361 studies identified in our PubMed search, 134 genome-wide transcriptomic studies in human gestational tissue samples were, based on a number of selection criteria, deemed eligible for systematic review (Additional file 1) [16–133]. These 134 studies were identified from a total of 116 distinct publications; this is so because 14 publications reported multiple comparisons that were separated into 33 distinct studies for the purpose of this analysis. Platform-wise, 127/134 (95 %) were microarray studies, 4/134 (3 %) were bisulfite-sequencing studies, and 3/134 (2 %) were RNA-sequencing studies. All studies were published between 1999 and 2014, primarily in the journals *Placenta* and *The American Journal of Obstetrics and Gynecology*. The phenotypes examined in these studies were quite diverse; 14/134 (10 %) studies examined preterm pregnancies, 80/134 (60 %) term pregnancies, and 40/134 (30 %) both preterm and term pregnancies. One non-clinical phenotype (healthy pregnancies) and 28 distinct clinical phenotypes were represented. Finally, 21/134 (16 %) were microRNA studies, 20/134 (15 %) were methylation studies, and the remaining 93/134 (69 %) were gene expression studies. A total of 10,993 unique genetic elements were reported to be transcriptionally active across all 134 studies (Additional file 2), but only 23/10,993 (0.2 %) were reported in 10 or more studies.

**Fig. 2:**
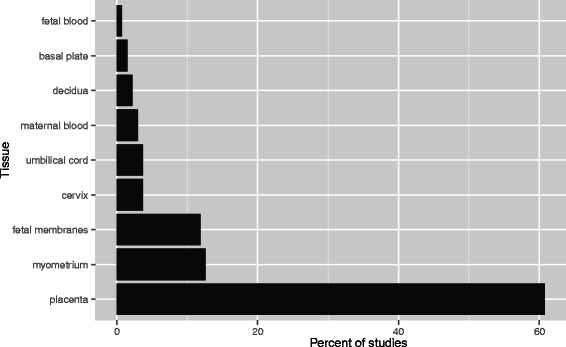
The vast majority of genome-wide transcriptomic studies on gestational tissues have focused on the placenta. A targeted PubMed search for genome-wide transcriptomic studies yielded a total of 134 studies focusing on 9 distinct gestational tissue types. Placental research accounted for 61 % of all studies in the meta-analysis, followed by fetal membranes (12 %) and myometrium (12 %)

The 134 studies analyzed 9 distinct gestational tissues, namely placenta, decidua, myometrium, maternal blood, cervix, fetal membranes (chorion and amnion), umbilical cord, fetal blood, and basal plate. The three most common tissues studied were placenta (82/134; 61 %), fetal membranes (16/134; 12 %), and myometrium (17/134; 12 %), whereas each of the other six tissues was sampled in 7 or fewer studies (Fig. 2).

**Fig. 3:**
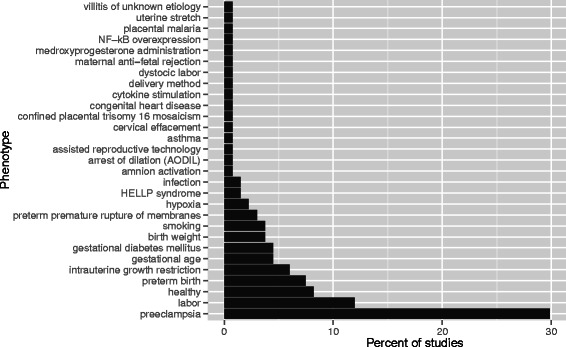
Gestational tissue transcriptomic studies in term and preterm human pregnancies organized by phenotype. A targeted PubMed search for genome-wide transcriptomic studies yielded a total of 134 studies focusing on 29 distinct phenotypes. PE research accounted for 30 % of all studies in the meta-analysis, followed by labor (12 %) and sPTB (7 %). Phenotype definitions are provided in Supplementary Table S2

The 134 studies analyzed 29 distinct phenotypes (Fig. 3). 11/134 (8 %) studies focused on healthy pregnancies, while the remaining 123/134 (92 %) studies focused on clinical phenotypes. The most common phenotypes studied were PE (40/134; 30 %), labor (16/134; 12 %), and sPTB (10/134; 7 %). Definitions for all phenotypes are provided in Additional file 3.

### PTB research focus does not reflect PTB subtype epidemiological prevalence

**Fig. 4:**
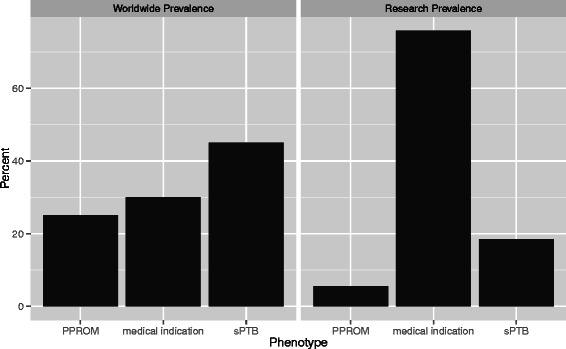
Proportion of transcriptomic research does not correspond to PTB subtype prevalence. Although only 30 % of all PTB cases are due to medical indications, such as PE, IUGR, or GDM, 76 % of the preterm studies in our systematic review focused on them. In contrast, only 18 % of the studies focused on sPTB, even though this clinical subtype accounts for the majority (45 %) of PTB cases

To evaluate whether the proportion of transcriptomic studies devoted on different PTB subtypes reflects their clinical prevalence, we compared the frequencies of the three major clinical etiologies (sPTB at 45 %, PPROM at 25 %, and medically indicated PTB at 30 %) to the frequency of transcriptomic studies devoted to these etiologies (Fig. 4). We found that although only 30 % of all PTB cases are due to medical indications, such as PE, IUGR, or GDM, 41/54 (76 %) of the studies categorized as preterm in our systematic review focused on them; 21/54 (39 %) of the preterm studies focused on PE alone. In contrast, although sPTB is responsible for 45 % of all cases, only 10/54 (18 %) of the preterm studies in our systematic review studied this clinical subtype.

### A meta-analysis of 93 gene expression studies across 9 distinct gestational tissues showed limited overlap of candidate genes

**Fig. 5:**
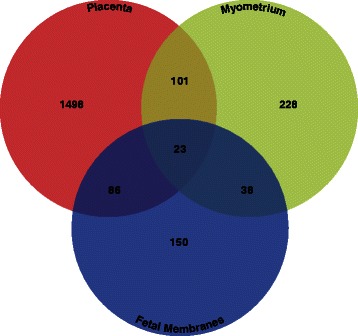
Overlap of differentially expressed genes across tissues. Differentially expressed genes present in two or more gene expression studies categorized by tissue were compared across the three most commonly studied (placenta, myometrium, and fetal membranes). Out of 2,123 genes identified to be differentially expressed in at least two studies, 23 genes were shared across all three tissues

To perform an aggregated meta-analysis, we focused on the 93/134 gene expression studies. These 93 gene expression studies analyzed all 9 distinct gestational tissues, namely placenta, decidua, myometrium, maternal blood, cervix, fetal membranes (chorion and amnion), umbilical cord, fetal blood, and basal plate. The three most common tissues studied for differential gene expression were placenta (53/93; 57 %), myometrium (17/93; 18 %), and fetal membranes (11/93; 12 %), whereas each of the other six tissues was sampled in 4 or fewer studies. Genome-wide gene expression profiling studies of the three most commonly studied gestational tissues, i.e., placenta, myometrium, and fetal membranes, identified a total of 8,437 unique differentially expressed genes, of which only 2,123 (25 %) were found in two or more studies (Fig. 5, Additional file 4). This examination also showed that only 23 candidate genes were differentially expressed two or more times in studies of all three tissues (Additional file 5). Among the genes present in this overlap were interleukin 1 beta, a proinflammatory cytokine shown to be involved in infection-related PTB and PE, and superoxide dismutase 2, an antioxidant enzyme shown to be involved in oxidative stress associated with PTB [18, 23, 34, 49, 65, 134–138].

### Although gene expression profiles are available for 29 distinct phenotypes, PTB research is dominated by studies focused on select phenotypes of PTB

**Fig. 6:**
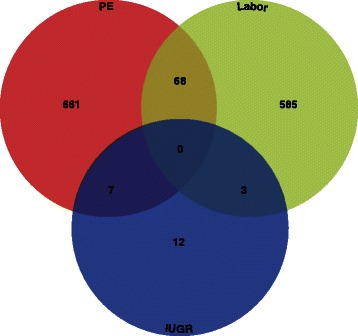
Overlap of differentially expressed genes across phenotypes. Differentially expressed genes identified in two or more gene expression studies categorized by phenotype were compared across the most commonly studied (PE, labor, and IUGR). Out of 1,336 genes identified to be differentially expressed in at least two studies, none were shared across all three phenotypes

The 93 gene expression studies analyzed 29 distinct phenotypes. From these studies, 5/93 (5 %) focused on a non-clinical phenotype (healthy pregnancies), with the remaining 88/93 (95 %) focused on clinical phenotypes. Among studies focused on clinical phenotypes, the three most common phenotypes investigated were PE (27/93; 29 %), labor (15/93; 16 %), and IUGR (8/93; 9 %); each of the other 26 clinical phenotypes was studied in 5 or fewer studies. Genome-wide gene expression studies of the three most commonly studied clinical phenotypes identified a total of 7,730 unique genes, of which only 1,336 (15 %) were present in two or more studies (Fig. 6, Additional file 6). No candidate genes were found two or more times in studies of all three phenotypes. Generally, overlap of differentially expressed genes was more limited across clinical phenotypes than across gestational tissues.

### Overlap of differentially expressed genes identified across PTB studies is limited

**Fig. 7:**
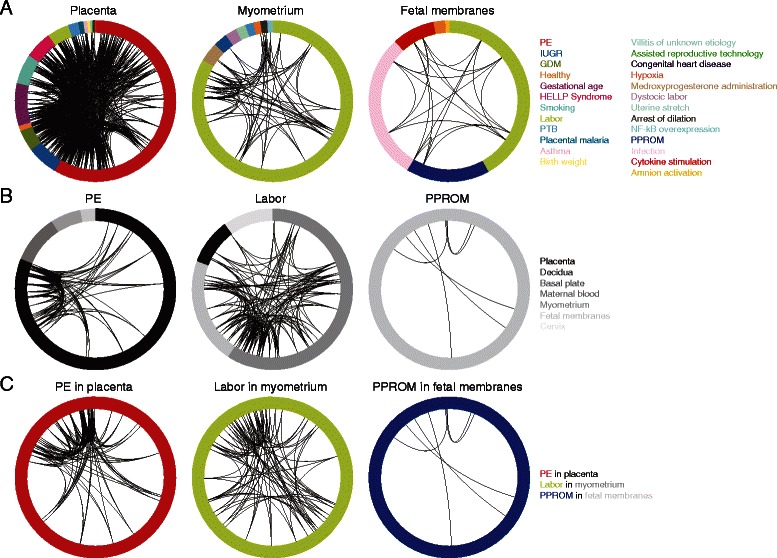
Representation of overlap in differentially expressed genes across the most commonly studied tissues, phenotypes, and tissues & phenotypes. Studies are represented as distinct wedges in the outermost track, colored by phenotype and sized by number of genes reported. Genes that show a high degree of overlap across studies (4 or more placenta, PE, *or* PE in placenta studies; 4 or more myometrium, labor, *or* labor in myometrium studies; 4 or more fetal membranes studies; or 2 or more PPROM *or* PPROM in fetal membranes studies) appear as black links connecting each study reporting the gene. In general, the scarcity of links illustrates the considerable lack of overlap in the genes identified as differentially expressed across PTB studies. **a** Representation of overlap in differentially expressed genes across the most commonly studied tissues. Studies of placenta, myometrium, and fetal membranes, the three most commonly studied tissues, focused on a total of 25 distinct phenotypes with PE dominating placental research, labor dominating myometrial research, and PPROM dominating fetal membranes research. **b** Representation of overlap in differentially expressed genes across the most commonly studied phenotypes. PE was studied across 4 distinct gestational tissues (placenta, decidua, basal plate, and maternal blood), labor was studied across 4 distinct gestational tissues (myometrium, fetal membranes, placenta, and cervix), and PPROM was studied across only 1 distinct gestational tissue (fetal membranes). **c** Representation of overlap in differentially expressed genes across the most commonly studied tissue *and* phenotype combinations. The most studied combinations were PE in placenta (n = 23), labor in myometrium (n = 9), and PPROM in fetal membranes (n = 3). Examination of PE in placenta studies identified 16 genes that were present in 4 or more studies, examination of labor in myometrium studies identified 15 genes that were present in 4 or more studies, and examination of PPROM in fetal membranes studies identified 6 genes that were present in 2 or more studies

Studies of placenta, myometrium, and fetal membranes, the three most commonly studied tissues, focused on a total of 25 distinct phenotypes (Fig. 7a, Additional file 7). The clinical phenotype studied, however, differed between tissues, with PE dominating placental research (23/53 placental studies or 43 %), labor dominating myometrial research (9/17 myometrial studies or 53 %), and PPROM dominating fetal membrane research (4/13 fetal membrane studies or 31 %). Likewise, the range of tissues studied differed between phenotypes. PE was studied across 4 distinct gestational tissues (placenta, decidua, basal plate, and maternal blood), labor was studied across 4 distinct gestational tissues (myometrium, fetal membranes, placenta, and cervix), and PPROM was studied across only 1 distinct gestational tissue (fetal membranes) (Fig. 7b, Additional file 8).

To identify common differential gene expression signatures, we looked for overlap between differentially expressed genes reported in studies of the same phenotype *and* tissue. The most studied phenotype-tissue combinations were PE in placenta (n = 23), labor in myometrium (n = 9), and PPROM in fetal membranes (n = 4) (Fig. 7c, Table 1). Examination of PE in placenta studies identified 16 genes that were present in 4 or more studies including *LEP*, a fat-regulating hormone commonly shown to be differentially expressed in gestational tissues of women with PE and HELLP Syndrome, and *FLT1*, a growth factor known to be highly expressed in preeclamptic placental trophoblast cells [21, 32, 44, 48, 53, 75, 80, 88, 94]. Examination of labor in myometrium studies identified 15 genes that were present in 4 or more studies including *PTGS2*, a cyclooxygenase involved in inflammation and commonly upregulated in myometrium during labor [18, 26, 40, 64, 66, 136, 139]. Finally, 6 genes were present in 2 or more PPROM in fetal membranes studies including *IL8*, a proinflammatory chemokine often associated with PTB [36, 37, 55, 87, 92, 140].

**Table 1:** The most often recovered differentially expressed genes in PE in placenta, labor in myometrium, and PPROM in fetal membranes

PE in placenta		
Entrez gene ID	Official gene symbol	# studies
3952	*LEP*	7
2321	*FLT1*	6
3623	*INHA*	6
3624	*INHBA*	6
2022	*ENG*	5
6647	*SOD1*	5
10148	*EBI3*	5
604	*BCL6*	4
1082	*CGB*	4
3972	*LHB*	4
10272	*FSTL3*	4
10544	*PROCR*	4
54210	*TREM1*	4
60676	*PAPPA2*	4
93659	*CGB5*	4
94115	*CGB8*	4
Labor in myometrium		
Entrez gene ID	Official gene symbol	# studies
165	*AEBP1*	4
366	*AQP9*	4
861	*RUNX1*	4
2354	*FOSB*	4
3164	*NR4A1*	4
3576	*IL8*	4
3976	*LIF*	4
5054	*SERPINE1*	4
5292	*PIM1*	4
5334	*PLCL1*	4
5743	*PTGS2*	4
6401	*SELE*	4
9123	*SLC16A3*	4
51129	*ANGPTL4*	4
117247	*SLC16A10*	4
PPROM in fetal membranes		
Entrez gene ID	Official gene symbol	# studies
972	*CD74*	2
1117	*CHI3L2*	2
3576	*IL8*	2
7805	*LAPTM5*	2
6280	*S100A9*	2
23574	*PRG1*	2

To examine whether the sets of genes that were most prevalent in each of the three tissue and phenotype pairs (PE in placenta, labor in myometrium, and PPROM in fetal membranes) disproportionally represented particular functions, we examined whether any Gene Ontology functional category was statistically significantly enriched (*p* < 0.0001) in each of the three gene sets (Additional file 9). Candidate genes identified in PE in placenta studies were enriched for regulation of cell death (GO:0010941) and apoptosis (GO:0042981), candidate genes identified in labor in myometrium were enriched for wounding (GO:0009611) and inflammatory response (GO:0006954), and candidate genes identified in PPROM in fetal membranes were enriched for immune system process (GO:0002376) and immune response (GO:0006955).

## Discussion

PTB is a complex, multifactorial syndrome with high prevalence worldwide, whose pathogenesis remains poorly understood, especially for cases of early spontaneous labor. To provide an overview as well as a synthesis of the current landscape of PTB transcriptomics, we conducted an in-depth systematic review of the literature as well as a meta-analysis of 93 gene expression studies on a wide diversity of gestational tissues and clinical phenotypes. Examination of our results identifies two key findings. First, the correspondence between PTB subtype prevalence and proportion of transcriptomic research devoted to these subtypes is weak. Second, the overlap between differentially expressed genes identified in different studies is quite small, even on studies aimed on the same phenotypes and tissues. Below, we discuss the possible factors that underlie these two key findings and their implications for research on PTB.

In general, transcriptomic studies on placental tissue samples from women with preeclampsia dominate PTB research. Furthermore, there are very few studies focusing on sPTB, a subtype responsible for 45 % of all PTB cases. Although genes commonly associated with PTB clinical subtypes (i.e., *LEP* and *FLT1*) are identified in many of the gene expression studies to be differentially expressed, the overlap between the differentially expressed genes identified across studies is generally very limited. This is not surprising in comparisons between tissues (Fig. 5) because these often involve examinations of different clinical subtypes, although it does suggest that there is little overlap in tissue-specific transcriptional profiles of different clinical subtypes. Similarly, it is not surprising that comparisons between clinical subtypes do not show a high degree of overlap (Fig. 6) because these often involve examinations of different tissues. Nevertheless, it should be noted that differentially expressed genes with substantial overlap across studies appear to be biologically meaningful. For example, genes involved in hormone regulation (i.e., *CGB*, *CRH*, *INHA*, and *GH2*), which have been previously shown to be key in the maintenance of pregnancy, show substantial overlap in preeclampsia studies. Genes involved in inflammation (i.e., *IL8*), which have been previously shown to be dysregulated in PPROM and other clinical PTB subtypes, are also identified to be differentially expressed in multiple studies.

The observed minimal overlap between the differentially expressed genes identified across studies focused on the same tissue *and* clinical phenotype (Fig. 7) is possibly more serious. One potential explanation may be the difficulty in obtaining appropriate controls important in pregnancy research; comparing studies that differ with respect to the presence of labor, gestational age, and fetal sex is challenging, since all of these factors are thought to influence the gene expression landscape in gestational tissues. Even though matching of samples with respect to all these factors is very challenging, the reporting of a standard list of such factors as required metadata in transcriptomic studies would facilitate further examination of their importance and likely influence on transcriptomic profiles.

In addition to transcriptomics, several other systematic reviews and meta-analyses have focused on identifying biomarkers, usually proteins, that are associated with PTB [141–143]. Overlapping 19 previously identified common PTB biomarkers with the studies in our meta-analysis indicates that most (12/19; 63 %) are replicated in 4 or more studies (Table 2). Therefore, our comparison shows evidence of considerable overlap between transcriptomic and proteomic studies in PTB. Further research from both approaches is necessary, however, because our comparison also indicates that transcriptomics and proteomics can target unique candidate genes and proteins as well.

**Table 2:** Overlap of meta-analysis with previously identified PTB biomarkers

Entrez gene ID	Official gene symbol	# studies
1392	*CRH*	11
6279	*S100A8*	10
3569	*IL6*	9
1082	*CGB*	9
2335	*FN1*	9
3553	*IL1B*	9
7124	*TNF*	6
5617	*PRL*	5
2810	*SFN*	4
6283	*S100A12*	4
2512	*FTL*	4
4318	*MMP9*	4
5443	*POMC*	2
174	*AFP*	1
4317	*MMP8*	0
308	*ANXA5*	0
3700	*ITIH4*	0
3558	*IL2*	0
6013	*RLN1*	0

Furthermore, the recent publication of comprehensive phenotyping tools necessitates the connection of evidence-based phenotype knowledge with genomic data collection in order to make more targeted conclusions [144]. It’s challenging to compare and contrast gene expression signatures between distinct subtypes without knowing whether the transcriptomes came from cases of sPTB due to maternal stress, uterine distention, or another subtype. Therefore, a greater focus needs to be placed on collecting the most detailed meta-data available regarding sPTB diagnosis as well as performing genome-wide studies of these newly described sPTB subtypes.

Finally, it is important to note that different studies follow different guidelines with respect to data availability. For example, some studies do not report the full list of differentially expressed genes identified or do not make them easily available for subsequent analysis (e.g., reporting tables that contain differential expression data on hundreds or thousands of genes in PDF format), therefore limiting and biasing the data available for subsequent analyses. The publishing of the data for *all* genes with differential expression above an explicit significance threshold in an easily accessible format is crucial in order to carefully analyze aggregated results and draw meaningful conclusions.

## Conclusions

This study synthesizes all high-quality transcriptomic studies on gestational tissues to examine the landscape of PTB as well as to identify genes and genomic elements associated with it. We found that highly prevalent PTB subtypes, such as sPTB, are not well studied and that differentially expressed genes identified in different studies are often non-overlapping. Thus, the identification of the genes whose dysregulation contributes to this complex and multifactorial syndrome will require many more large-scale, systematic studies aimed at understanding the transcriptional profiles of these diverse clinical PTB subtypes across gestational tissues and characterizing their disease-relevant transcriptional differences.

### Note Added in Proof

While this manuscript was in review, by studying the variation in the placental transcriptome of healthy humans, Hughes and coworkers estimated that more than 90 % of the observed transcriptomic variation is explained by variation within and between individuals [145]. These results provide an alternative, yet complementary, explanation for our finding that profiles of differentially expressed genes were highly heterogeneous both between and within clinical subtypes and tissues as well as between studies of the same clinical subtype and tissue.

## Methods

### Search strategy

This systematic review and meta-analysis followed guidelines set by the Preferred Reporting Items for Systematic Reviews and Meta-Analyses (PRISMA) (Additional files 10, 11 and 12) [146]. The electronic search was performed on August 16, 2014 in PubMed with no restrictions to identify all articles relating to differentially expressed or methylated genes and microRNAs in human gestational tissues. The search strategy was constructed based on related MeSH terms:

*“Pregnancy”[mh] AND “Humans”[mh] AND (“Gene Expression Profiling”[mh] OR “Gene Expression Regulation”[mh]) AND (“Placenta”[mh] OR “Decidua”[mh] OR “Myometrium”[mh] OR “Cervix Uteri”[mh] OR “Extraembryonic Membranes”[mh] OR “Blood”[mh] OR “Plasma”[mh] OR “Umbilical Cord”[mh])*

### Systematic review

We collected abstracts for all 2,361 studies identified from this search and annotated eligibility based on 6 inclusion criteria:

1. Published in English
2. Full text available
3. Original research
4. Human gestational tissue samples
5. Genome-wide analysis
6. Candidate gene list assembled

134 studies met all 6 criteria and were included in the systematic review. Furthermore, studies were excluded when the study data was not accessible (the number of gene candidates was reported but the list of candidate genes was not), the study data was not reported (the number of candidate genes was not reported and a list of candidate genes was not provided), the data was unclear, there were no significant gene candidates, the study was not genome-wide, the study was not human-specific, the study was not relevant, the study was not single-gene based (i.e., was focused on pathways or gene sets), the study used data from proteomics, the study was performed on cell line rather than in an in-vivo tissue, the study’s supplement was not available, or when the study’s tissue was collected before the third trimester (Additional file 12).

### Meta-analysis

Studies were included in our meta-analysis if they met an additional 3 inclusion criteria:

1. Studied differential gene expression
2. Provided candidate gene list
3. DAVID ID conversion successful

116 references met all inclusion criteria and, due to multiple comparisons or analyses in 14 of these references, a total of 134 distinct studies were summarized (Additional file 1). Of the 134 studies included in our systematic literature review, 93 gene expression studies met these criteria and were further analyzed. All differentially expressed genes reported in these studies were first extracted and then converted to Entrez ID format using the DAVID online tool, selecting the smallest Entrez ID number if multiple IDs mapped to single genes. We extracted all reported significantly differentially expressed genes based on each study’s significance threshold for differential expression. Overlap was determined simply by the presence of the same gene in the gene lists from different studies. DAVID was used to assay functional enrichment according to Gene Ontology categories. All analyses were performed using Python and visualizations were performed using ggplot2 and Circos [147, 148].

## Additional files

Additional file 1:
**Summary of studies in systematic review.**
Additional file 2:
**All reported candidate genomic elements.**
Additional file 3:
**Phenotype definitions.**
Additional file 4:
**Duplicated genes in well-studied gestational tissues.** Genes in 2 or more placenta studies *or* 2 or more myometrium studies *or* 2 or more fetal membranes studies. Additional file 5:
**Well-replicated genes in well-studied gestational tissues.** 22 genes in 2 or more placenta studies *and* 2 or more myometrium studies *and* 2 or more fetal membranes studies. Additional file 6:
**Duplicated genes in well-studied clinical phenotypes.** Genes in 2 or more PE studies *or* 2 or more labor studies *or* 2 or more IUGR studies. Additional file 7:
**Well-replicated genes in placenta, myometrium, and fetal membranes.** Genes in 5 or more placenta studies *or* 5 or more myometrium studies *or* 5 or more fetal membranes studies. Additional file 8:
**Well-replicated genes in PE, labor, and PPROM.** Genes in 5 or more PE studies *or* 5 or more labor studies *or* 2 or more PPROM studies. Additional file 9:
**GO enrichment.** Enriched GO functional categories for replicated genes in PE in placenta, labor in myometrium, and PPROM in fetal membranes. Additional file 10:
**PRISMA checklist.**
Additional file 11:
**PRISMA flow chart.**
Additional file 12:
**Excluded studies.** All studies excluded from systematic review.

## Abbreviations

PTB: Preterm birth
sPTB: Spontaneous idiopathic preterm birth
PE: Preeclampsia
IUGR: Intrauterine growth restriction
GDM: Gestational diabetes mellitus
PPROM: Preterm premature rupture of membranes
MeSH: medical subject headings
