# Supplementary material for: Gestational tissue transcriptomics in term and preterm human pregnancies: a systematic review and meta-analysis
Source: BMC Med Genomics. 2015 Jun 5;8:27. doi: 10.1186/s12920-015-0099-8 (PMC4456776; doi:10.1186/s12920-015-0099-8)
Supplement: Additional file 11: — PRISMA flow chart. [file 12920_2015_99_MOESM11_ESM.doc]

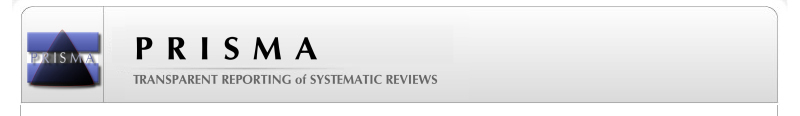
**PRISMA 2009 Flow Diagram**

**Screening**

**Included**

**Eligibility**

**Identification**

Records identified through database searching
(n = 2,361)

Additional records identified through other sources
(n = 0)

Records after duplicates removed
(n = 2,361)

Records screened
(n = 2,361)

Records excluded
(n = 2,223)

Full-text articles assessed for eligibility
(n = 237)

Full-text articles excluded, with reasons
(n = 101)

Studies included in qualitative synthesis
(n = 134)

Studies included in quantitative synthesis (meta-analysis)
(n = 93)
